# Evaluation of a rapid identification panel for fungemia

**DOI:** 10.1093/jacamr/dlaf110

**Published:** 2025-06-26

**Authors:** Emily A Siegrist, Bryan P White, Denise Robison, Cindy McCloskey, Maria Alkozah, Nelson Agudelo Higuita, Rita Wilson Dib, Joseph Sassine

**Affiliations:** Department of Pharmacy, OU Health, 700 NE 13th Str., Oklahoma City, OK 73104, USA; Department of Pharmacy, OU Health, 700 NE 13th Str., Oklahoma City, OK 73104, USA; Department of Pathology, OU Health, P.O. Box 26901, BMSB 451, Oklahoma City, OK 73126-0901, USA; Department of Pathology, OU Health, P.O. Box 26901, BMSB 451, Oklahoma City, OK 73126-0901, USA; Department of Medicine, Infectious Diseases Section, OU Health Sciences Center, 800 Stanton L Young Blvd, Oklahoma City, OK 73104, USA; Department of Medicine, Infectious Diseases Section, OU Health Sciences Center, 800 Stanton L Young Blvd, Oklahoma City, OK 73104, USA; Department of Medicine, Infectious Diseases Section, OU Health Sciences Center, 800 Stanton L Young Blvd, Oklahoma City, OK 73104, USA; Department of Medicine, Infectious Diseases Section, OU Health Sciences Center, 800 Stanton L Young Blvd, Oklahoma City, OK 73104, USA

## Abstract

Bloodstream infections due to yeast are associated with a high mortality rate. There is a lack of data that evaluate the real-world sensitivity of a rapid detection system for bloodstream infections due to yeast or the impact of these results on antimicrobial stewardship. The aim of this study was to evaluate the sensitivity of an ePlex panel (BCID-FP) for rapid detection of yeast from a positive blood culture bottle and to evaluate the impact of these rapid results on antifungal escalation or de-escalation. We evaluated 63 episodes of fungemia and found a sensitivity of 94%, lower than the 99%–100% stated in the package insert. Most common pathogens were *Candida glabrata* (36%), *Candida albicans* (24%), *Candida parapsilosis* (10%) and *Candida krusei* (10%). Only 57.1% of BCID-FP results lead to a change in antifungal therapy, most of which was initiation of an echinocandin. The real-world sensitivity of the BCID-FP panel was lower than anticipated and rarely led to de-escalation of antifungal therapy.

## Introduction

*Candida* bloodstream infections are among the most common bloodstream infections in hospitalized patients and are associated with a high mortality rate.^1^ Blood culture remains the mainstay of diagnosis for candidiasis, but the poor sensitivity and slow turnaround time can negatively affect patient care.^2^ The Cobas^®^ ePlex BCID-FP^®^ (GenMark Diagnostics, CA, USA, currently rebranded as Roche Cobas^®^ ePlex) is a multiplex microarray assayed on blood cultures that identifies 10 *Candida* species as well as *Cryptococcus*, *Rhodotorula* and *Fusarium* spp. (Table 1). The BCID-FP panel detects individual *Candida* organisms, not all organisms within a group (e.g. *Candida parapsilosis en strictu sensu*, not *Candida parapsilosis complex*). This panel provides results in 90 minutes, significantly faster than traditional culture-based methods, with a reported sensitivity and specificity of 100% for many *Candida* species.^2,3^

**Table 1.** GenMark yeast panel target organisms

Organism		
*Candida albicans*	*Candida kefyr*	*Cryptococcus gattii*
*Candida auris*	*Candida krusei*	*Cryptococcus neoformans*
*Candida dubliniensis*	*Candida lusitaniae*	*Fusarium*
*Candida famata*	*Candida parapsilosis*	*Rhodotorula*
*Candida guilliermondii*	*Candida tropicalis*	

There are limited data examining the sensitivity of the BCID-FP panel in real-world settings. In addition, its role in antifungal stewardship activities and impact on patient outcomes need to be better understood. The focus of this study was to evaluate the utility and performance of the BCID-FP panel.

## Patients and methods

We evaluated all admitted patients with a yeast isolated in the blood and an BCID-FP panel result between July 2023 and July 2024. Blood cultures were incubated in standard aerobic and anaerobic bottles on BD BACTEC^TM^ FX. Positive blood cultures were Gram stained and BCID-FP was conducted in parallel with standard laboratory culture. Identification of culture was done using MALDI-TOF MS (Bruker) and susceptibility testing was done using Sensititre^TM^ YeastOne panels. Duplicate fungemia episodes were included if a patient had multiple episodes separated by at least 7 days. Polymicrobial blood cultures were defined as the growth of multiple organisms in the same set of blood cultures that had the positive BCID-FP. Positive blood cultures are called to an on-call stewardship pharmacist 0700–1600.

## Results

We found 63 episodes of fungemia from 62 unique patients (Table 2). The most common pathogens were *Candida glabrata* (36%), *Candida albicans* (24%), *C. parapsilosis* (10%) and *Candida krusei* (10%) and the most common source was intra-abdominal (35%). All *Candida* spp. were susceptible to micafungin, but susceptibility to fluconazole was variable (Table 2). The BCID-FP panel resulted at a median of 2.1 hours (IQR 2–2.4) from the time of the Gram stain result, at a median of 50.7 hours (IQR 39.4–59.7) before the final culture result. Antifungals were initiated before blood culture collection in 11 patients (17.4%). Five of the 63 episodes (7.9%) were not identified on the BCID-FP panel, of which one was *Candida orthopsilosis*, an off-panel organism. Thus, the overall sensitivity for on-panel organisms was 94%. The four on-panel organisms that the BCID-FP panel did not detect were *C. glabrata* (one), *Candida lusitaniae* (one), *Candida dubliniensis* (one) and *C. parapsilosis* (one). Sensitivity of the BCID-FP panel may have been affected by polymicrobial growth, which was present in two blood culture sets that were not identified or by antifungal initiation prior to blood culture collection, which occurred in two of the episodes that were not detected by the panel.

**Table 2.** Baseline demographics and infection characteristics

Characteristic (*N* = 62)	*N* (%)
Age category	
Adult	54 (87)
Paediatric	8 (13)
Age in years, median (IQR)	57 (38.5–65.5)
Primary service (*N* = 63)	
Medical ICU	19 (30)
Internal medicine/hospital medicine/family medicine	18 (28)
Paediatrics/paediatric ICU	8 (12)
Surgical ICU	5 (8)
Transplant	4 (6)
Oncology/BMT	3 (5)
Surgical subspecialties	7 (11)
Source (*N* = 63)	
Central venous catheter	19 (30)
Intra-abdominal	22 (35)
Urine	7 (11)
Bone/joint	3 (5)
CNS	1 (2)
Other/multiple	11 (17)
Organism detected on BCID-FP (*N* = 63)	
*Candida glabrata*	23 (36)
*Candida albicans*	15 (24)
*Candida parapsilosis*	6 (10)
*Candida krusei*	6 (10)
*Candida tropicalis*	4 (6)
*Candida dubliniensis*	2 (3)
*Candida lusitaniae*	1 (1.5)
*Cryptococcus neoformans*	1 (1.5)
No targets detected	5 (8)
Antifungal at time of blood draw (*N* = 63)	11 (17.4)
Micafungin	6/11 (54.5)
Fluconazole	4/11 (36.4)
Isavuconazole	1/11 (9.1)
Fluconazole susceptibility by organism final ID, % susceptible (MIC_50_/MIC_90_)	
*Candida albicans* (*N *= 15)	100% (0.5/4)
*Candida dubliniensis* (*N* = 3)	N/A (0.25/0.5)
*Candida glabrata* (N = 24)	83% (4/32)
*Candida krusei* (*N* = 6)	N/A (assumed intrinsic resistance)
*Candida lusitaniae* (*N* = 2)	N/A (1/2)
*Candida orthopsilosis* (*N* = 1)^a^	N/A (0.5)
*Candida parapsilosis* (*N* = 7)	100% (0.25/0.5)
*Candida tropicalis*^b^ (*N* = 4)	25% (4/4)

^a^Only one organism tested with MIC 0.5.

^b^ *Candida tropicalis* during this time was tested on Sensititre YeastOne panels, which have a known limitation of false elevation in fluconazole MICs.

In 27 episodes (42.9%), the BCID-FP result did not lead to a change in antifungal therapy: 25 of these were receiving appropriate empirical antifungal therapy that was mostly an echinocandin and two elected not to escalate therapy due to goals of care. In 36 episodes (57.1%), the patient was initiated on an antifungal after the positive BCID-FP result: 34 with micafungin, one with voriconazole and one with fluconazole. The BCID-FP panel adds ∼$125 in laboratory costs for each patient, not including technician time or initial instrument purchase and validation.

## Discussion

We found significantly lower sensitivity when using the BCID-FP panel (94% versus 99%–100%) than was anticipated on the basis of product labelling. This difference in positivity is potentially due to the use of used many contrived samples cited in the device package insert. In these validations, a known isolate was inoculated into a blood culture bottle and may not reflect real-world samples and conditions.^3^ Further, limits of detection for positive blood culture bottles on positive blood culture have only been validated in blood culture bottles positive for single specimens. Mixed cultures may have quantities of *Candida* below the level of detection of the panel. Furthermore, actual clinical isolates may have different sequence variants leading to negative results.^3^ This study is limited by low sample size and small diversity of species tested, which may have affected our sensitivity. Further study is needed with larger and diverse samples to validate these findings.

Data supporting the use of echinocandins as the initial antifungal therapy for candidaemia have shown improved clinical success^4^ and in a meta-analysis, lower mortality compared to azoles.^5^ Due to a high prevalence of non-*albicans Candida* spp. in our centre, micafungin remains our preferred treatment for candidaemia, even after the BCID-FP panel results are available. BCID-FP results did not lead to antifungal de-escalation, and we observed low rates of fluconazole use early in patients with candidaemia. The BCID-BP panel may be useful for antifungal escalation at institutions that preferentially use fluconazole as their empirical antifungal, or that have lower rates of non-*albicans* candidaemia. However, the laboratory costs of the panel should be weighed against antifungal spending with careful evaluation of the impact on treatment decisions and patient outcomes. In conclusion, the BCID-FP was less sensitive than previously reported in the literature and, at our institution, rarely led to de-escalation of antifungals.

## References

[1] CDC . Data and Statistics on Candidemia. https://www.cdc.gov/candidiasis/data-research/facts-stats/index.html (date last accessed 10 February 2025).

[2] Chastain DB, White BP, Tu PJ et al Candidemia in adult patients in the ICU: a reappraisal of susceptibility testing and antifungal therapy. Ann Pharmacother 2024; 58: 305–21. 10.1177/10600280231175201 37272474

[3] ePlex Blood Culture Identification Fungal Pathogen Panel Package Insert. Clinical Micro Sensors, Inc. dba GenMark Diagnostics, 2020.

[4] Kullberg BJ, Viscoli C, Pappas PG et al Isavuconazole versus caspofungin in the treatment of candidemia and other invasive *Candida* infections: the ACTIVE trial. Clin Infect Dis 2019; 68: 1981–9. 10.1093/cid/ciy827 30289478

[5] Andes DR, Safdar N, Baddley JW et al Impact of treatment strategy on outcomes in patients with candidemia and other forms of invasive candidiasis: a patient-level quantitative review of randomized trials. Clin Infect Dis 2012; 54: 1110–22. doi:10.1093/cid/cis021 22412055

